# The pathogenesis of blepharospasm

**DOI:** 10.3389/fneur.2023.1336348

**Published:** 2024-01-11

**Authors:** Lixia Zhu, Hongmei Meng, Wuqiong Zhang, Wenjing Xie, Huaiyu Sun, Shuai Hou

**Affiliations:** ^1^Department of Neurology, The First Hospital of Jilin University, Changchun, China; ^2^Department of Neurology, The Second Hospital of Jilin University, Changchun, China

**Keywords:** dystonia, blepharospasm, pathogenesis, basal ganglia, cerebellum, neurotransmitters, dopamine, serotonin

## Abstract

Blepharospasm is a focal dystonia characterized by involuntary tetanic contractions of the orbicularis oculi muscle, which can lead to functional blindness and loss of independent living ability in severe cases. It usually occurs in adults, with a higher incidence rate in women than in men. The etiology and pathogenesis of this disease have not been elucidated to date, but it is traditionally believed to be related to the basal ganglia. Studies have also shown that this is related to the decreased activity of inhibitory neurons in the cerebral cortex caused by environmental factors and genetic predisposition. Increasingly, studies have focused on the imbalance in the regulation of neurotransmitters, including dopamine, serotonin, and acetylcholine, in blepharospasm. The onset of the disease is insidious, and the misdiagnosis rate is high based on history and clinical manifestations. This article reviews the etiology, epidemiological features, and pathogenesis of blepharospasm, to improve understanding of the disease by neurologists and ophthalmologists.

## 1 Introduction

Blepharospasm is the most common focal cranial dystonia. It is characterized by involuntary eyelid closure and effortful opening (1). Blepharospasm most commonly occurs in the orbicularis oculi muscles bilaterally, but in rare cases begins unilaterally (2).

The initial clinical symptoms of blepharospasm patients are mild and can be characterized by an increase in blinking or a desire to close their eyes totally, usually with slow progression. In advanced stages of the disease, persistent eyelid closure and even functional visual blindness may occur, seriously affecting the patient's work, life, and studies (3). Symptoms are often triggered or exacerbated by tension, anxiety, or fatigue, and can be relieved by specific movements, such as touching the face or eyelids, talking, singing, etc, which are called “sensory tricks” (1, 3, 4). Some studies have reported that more than half of patients with blepharospasm use one or more sensory tricks, most commonly touching of the eyelids, singing, humming, and talking (1, 5). Up to 87% of patients with blepharospasm possess one or more of these sensory skills (2, 6). In addition, spontaneous remission of blepharospasm is rare, occurring in < 10% of these patients, usually within the first 5 years of symptom onset (7).

The prevalence rates of blepharospasm vary greatly in different regions. A regional epidemiological study in the United States reported a range of 13–130 cases per million (8). The prevalence of primary focal dystonia in Europe in 2000 was 117 persons/million, with blepharospasm accounting for 36 persons/million (95% confidence interval 31–41 persons/million) (2, 9). However, because most epidemiological surveys are based on counting treated patients, the actual prevalence is likely to be higher in the population. Blepharospasm can be divided into primary blepharospasm (also known as idiopathic blepharospasm) and secondary blepharospasm, which commonly occurs secondary to cerebral hemorrhage, cerebral infarction, cerebrovascular malformation, multiple sclerosis, and delayed movement disorders (1).

At present, the pathogenesis of blepharospasm has not been fully elucidated. The disease pathogenesis involves susceptibility factors, anatomy and physiology, structural and functional imaging, and neurotransmitters. These factors do not act independently, but in concert. This review elaborates on the etiology and pathogenesis of primary blepharospasm with a view to improving the understanding of this disease by neurologists and ophthalmologists, from the point of view of these four aspects.

## 2 Susceptibility factors

Blepharospasm is more common in middle-aged and older patients, aged from 50 to 70 years, which indicates that age is an independent risk factor for this condition. The incidence is greater in women than in men, with a male:female ratio of ~1:2.3. People living in cities are at higher risk of the disease (10).

Blepharospasm is considered to be related to genetic susceptibility, environmental factors, and many other factors (11–14), but the exact cause has not been determined as yet (6, 15). Genes that increase the risk of blepharospasm include GNAL, TOR1A, CIZ1, and DRD5 (16). A study of 20 patients with blepharospasm in a Chinese population analyzed 151 genes associated with dyskinesia by means of next-generation sequencing. They found that seven patients had SYNE1 mutations, two patients each had CIZ1, LRRK2, CACNA1A, and FUS mutations, seven patients had mutations in two genes, and two patients did not have any mutations in the 151 genes analyzed. Only two patients had a family history of blepharospasm; mutations in SYNE1 and CIZ1 were detected in these patients, suggesting that mutations in SYNE1 and CIZ1 are the main genetic factors contributing to blepharospasm in these families (17). However, due to the small sample size in this study and the fact that other potential blepharospasm-related genes were not included, the role of genetic susceptibility factors in the pathogenesis of blepharospasm warrants further exploration.

A family history of dystonia or postural tremor, history of head and facial trauma with transient disturbance of consciousness, xerophthalmia (18, 19), history of eyelid inflammation or keratitis and sunlight exposure (20), etc., are also related to blepharospasm. Anxiety and depression were identified as independent risk factors of blepharospasm. Conversely, smoking, alcohol consumption, and coffee consumption may reduce the risk of developing blepharospasm (21, 22). A multicenter case–control study in Italy showed that coffee consumption was negatively correlated with the risk of primary blepharospasm, and that the intensity of this association increased with an increase in the average number of cups of coffee per day (23). Caffeine (24), one of the major components in coffee, antagonizes adenosine receptors in the cholinergic cells of the striatum, thus regulating basal ganglia dysfunction and alleviating blepharospasm symptoms.

## 3 Anatomy and physiology

Blepharospasm is a disease of dysfunction, and neurophysiology is a key step in research in this field. The blink reflex is an important component in the evaluation of patients with blepharospasm. The blink reflex consists of two parts: early ipsilateral R1 and late bilateral R2 (25). R1 is the pontine reflex, and R2 involves the more complex pontine and medulla oblongata reflexes. The R1 and R2 pathways in the blink reflex run from the trigeminal nerve (the fifth cranial nerve) through the trigeminal sensory nucleus and the facial nucleus to the facial nerve (the seventh cranial nerve). Based on electromyography, Berardelli et al. (26) found that electrical stimulation of the supraorbital nerve in blepharospasm patients prolonged the duration of the R2 part of the blink reflex and shortened the recovery cycle of the R2 part of the blink reflex, which indicated the existence of abnormalities in the neural pathway mediating the R2 response of the blink reflex in blepharospasm patients. Additionally, the blink reflex can be used to examine various functions regulated by the brainstem. Without interference from peripheral nerve damage, studying the transient reflex would be a useful technique for assessing supranuclear control of excitability in brainstem neurons (27).

### 3.1 Neural networks

Dystonia is associated with abnormal activity in multiple brain regions, including the basal ganglia, brainstem, cerebellum, motor cortex, and auxiliary motor areas (28, 29). Because multiple anatomical regions are associated with dystonia, it is increasingly recognized that dystonia is a neural network disease caused by dysfunction of one or more network nodes (30, 31). Similar to the pathogenesis of tremor, we propose two circuits for blepharospasm. For initiation, basal ganglia (putamen/striatum) are the central player along with their connection with the cerebellum (30). In terms of continuity, the cerebellar-thalamic-cortical network plays an increasingly important role.

#### 3.1.1 Basal ganglia

The basal ganglia are a group of gray matter nuclei located at the depths of both cerebral hemispheres and are the main structures of the extrapyramidal system. The basal ganglia are considered a classical site for explaining the pathogenesis of blepharospasm and other craniocerebral dystonias (32–34). Basal ganglia dysfunction can lead to the loss of inhibition of brain interneurons by the basal ganglia, resulting in increased excitability of the brainstem interneurons. Electromyographic (EMG) studies have found abnormalities in both the R1 wave amplitude and duration, as well as the R2 reflex duration and recovery, in blepharospasm (35). It has been also found that inhibition of the R2 period is decreased in patients with blepharospasm compared to healthy controls (36). These studies have demonstrated that the pathogenesis of blepharospasm originates from the increased excitability of brainstem interneurons, which may be caused by dysfunction of the basal ganglia (37, 38). A body of evidence suggests a central role for the basal ganglia, whose function is regulated by various neurotransmitters, in blepharospasm (39, 40). For example, Ferrazzano et al. found that an imbalance in neurotransmitter transmission in the basal ganglia of the brain may lead to abnormal eye performance in the blink reflex center of patients with blepharospasm (41, 42). A study found that decreased intracranial dopamine receptors in the basal ganglia of the skull, leading to impaired metabolic pathways of dopamine or other neurotransmitter systems, may be an important mechanism in the pathogenesis of blepharospasm (43). However, growing evidence suggests that blepharospasm cannot be explained exclusively by lesions in the basal ganglia.

In recent years, an increasing number of studies have shown that, in addition to basal ganglia dysfunction, blepharospasm may also be involved in dysfunction of a brain neural network involving the cerebellum, thalamus, globus pallidus, cerebral cortex, and other regions (33, 44–48). To locate the brain structures involved in blepharospasm, Girard et al. performed magnetic resonance imaging (MRI) in six patients with a history of cerebrovascular accident or head trauma. Studies have shown that blepharospasm is not caused by localized injury, but by neurophysiological changes in the cortex–striatum–globus pallidus–thalamus–cortex and cerebellar–thalamus–cortex circuits (34, 49, 50).

#### 3.1.2 Cerebellum

The cerebellum, located at the back of the fourth ventricle, is important for maintaining body balance and limb muscle tension. Recent evidence suggests that the cerebellum is involved in the pathogenesis of blepharospasm. Existing animal experimental data have suggested that the cerebellum affects the pathogenesis of dystonia through at least three mechanisms: abnormal cerebellar efferent signal patterns, abnormal connections between the cerebellar and basal ganglia nuclei, and abnormal morphology or structure of the cerebellar cells (51). Fagan et al. (52) compared a histopathological analysis of cerebellar tissue sections in seven patients with blepharospasm and nine controls. The density of cerebellar Purkinje neurons in patients was significantly lower than that in controls. This finding was essentially the same as that of Prudente et al. (53) in six cases of cervical dystonia, which indicated that the pathological anatomy of dystonia associated with different disease has something in common. Mascia et al. (48) hypothesized that neurophysiological abnormalities in blepharospasm may reflect abnormal activation and functional connectivity involving the frontal and parietal cortical layers, basal ganglia, thalamus, and cerebellum. The presence of an abnormal blink reflex recovery cycle in patients with blepharospasm confirms the hyperexcitability of the trigeminal facial circuit. A study on 30 patients with blepharospasm, and 20 sex- and age-matched healthy controls, found impaired prepulse inhibition of the blink reflex in patients with blepharospasm, suggesting that abnormal regulation of cortical and subcortical inhibition may also lead to hyperexcitability of the trigeminal facial circuit (54).

Neurophysiological studies have also shown that the cerebellum plays a role in the pathophysiology of dystonia (51); however, no conclusive evidence that the cerebellum is the main or only neuroanatomical origin of the disease exists at present (46).

### 3.2 Loss of inhibition

Loss of motor inhibition has been noted at the spinal cord, brainstem, and cortex, and is a definitive functional feature of dystonia (55, 56). There is also a study evaluating other spinal cord and brainstem inhibitory reflexes (such as transient and perioral reflexes) that confirm motor inhibitory processes are reduced in patients with primary dystonia when compared with the control group (57).

### 3.3 Abnormal sensorimotor integration

Previous studies have suggested that the integration of sensorimotor information is abnormal in dystonia (55). The role of impaired sensory processing is particularly important for dystonia (58). “Sensory tricks” can alleviate or ameliorate dystonic spasms, while controlling sensory inputs can trigger (muscle vibration) or relieve (muscle afferent blockad) dystonia (55). At the same time, impaired neuromotor control is observed in blepharospasm (59). Baker et al. (60) found that patients with blepharospasm had abnormally increased activation in the visual cortex, primary motor cortex (M1), limbic system, and cerebellum. Taken together, these studies suggest that sensory-motor integration is impaired in blepharospasm. Overall, evaluations of the sensory-motor integration system in dystonia have allowed for a substantial increase in understanding the phenomenology and pathogenesis of blepharospasm.

### 3.4 Abnormalities of synaptic plasticity

As the basis of learning and memory, synaptic plasticity is one of the most important characteristics of the central nervous system. A series of previous studies have suggested that abnormal synaptic plasticity is an important factor in the pathophysiological mechanism of dystonia (61–63). Importantly, Martella et al. (61) found that synaptic plasticity was abnormal in the striatum of a DYT1 dystonia mouse model constructed by transgenic technology. Indeed, deep brain stimulation and transcranial magnetic stimulation methods that explore mechanisms of synaptic plasticity in the brain motor cortex show a maladaptive plasticity of sensory-motor integration in patients with dystonia (64).

In summary, we conclude that the pathophysiological mechanisms of BSP mainly include the impairment of sensorimotor integration, abnormalities of synaptic plasticity, and decreased inhibition. Together, these mechanisms lead to an imbalance in the basal ganglia gating system, resulting in inadequate inhibition of noisy activity and a hyperactivation of cerebral cortical areas (29, 31).

## 4 Structural and functional imaging

Conventional MRI of blepharospasm revealed no obvious abnormalities. However, with the development of emerging neuroimaging techniques (65), the microstructure and functional changes of other brain regions have become visible, providing a new method for analyzing brain function and structure in patients with blepharospasm. For example, voxel-based morphometry (VBM) and diffusion tensor imaging (DTI) are used to evaluate structural changes in the brain, whereas functional magnetic resonance imaging (fMRI) and positron-emission tomography (PET) are used to evaluate functional changes.

### 4.1 Structural imaging

#### 4.1.1 VBM

VBM is an MRI technique used to compare the structural anatomy of patients and healthy controls. When interpreting the results of a VBM study, the location of the abnormality is of paramount importance. A comparative study of 62 patients and 62 age/sex matched healthy subjects found that, with an increase in the duration of blepharospasm, the volume of GM increased from the right supplementary motor area (SMA) to the cortical–basal ganglia motor and visual–motor integration pathways, suggesting that blepharospasm is associated with extensive GM structural abnormalities (66). Another meta-analysis, involving 129 patients with blepharospasm and 144 healthy controls, showed an increase in GM in the bilateral precentral and postcentral gyri, SMA, and bilateral paracenter lobules in patients with blepharospasm, which provides a new concept of blepharospasm as a type of brain network dysfunction (67).

#### 4.1.2 DTI

DTI is a non-invasive method that can effectively observe and evaluate the ultrastructure of WM. Numerous previous studies have reported abnormal GM structures in the basal ganglia, sensorimotor cortex, and cerebellum of patients with blepharospasm (66, 68). For example, Martino et al. (69) performed VBM on 25 patients with primary blepharospasm and on 24 healthy controls. The volume of the GM in the primary sensory cortex (S1) of patients with blepharospasm was decreased, which was consistent with functional defects in the same region. Their study suggested that the S1 region is a key cortical region in the pathophysiology of blepharospasm. As GM and WM are closely connected, we speculate that structural changes exist in the WM of patients with blepharospasm, particularly in the above-mentioned anatomical structures. However, the conclusions of DTI research are not completely consistent. Two DTI studies on patients with blepharospasm using the region-of-interest-based method (70) and the whole-brain method (71) found no significant change in fractional anisotropy (FA). However, Yang et al. (72) compared 20 blepharospasm patients with 11 healthy controls and found that the FA of the WM in the left cerebellum of blepharospasm patients was significantly lower than that of healthy controls and negatively correlated with disease severity, indicating that the cerebellum is an important area of the blepharospasm motor network model and that DTI is an important parameter for disability assessment in blepharospasm patients.

### 4.2 Functional imaging

#### 4.2.1 fMRI

fMRI using blood oxygen level-dependent imaging (73) is considered an important imaging method for studying changes in brain function in blepharospasm and is also the focus of current research. Using fMRI, Glickman et al. (74) found that the cerebellar–thalamic–cortical network correlated positively with the severity of blepharospasm, whereas the basal ganglia and cerebellar motor circuits were related to the trigger of blepharospasm. The putamen (25, 34, 74) may be the trigger of blepharospasm. Previous studies have also confirmed blepharospasm is triggered by activation of the putamen, whereas fMRI has shown that disinhibition in the striatum is the starting point of eyelid spasms (34).

#### 4.2.2 PET

PET can be used as a non-invasive measure of glucose metabolism in the brain based on positron tracking (75). PET studies conducted by Suzuki et al. showed hypermetabolism of glucose in the bilateral thalamus in patients with blepharospasm (76). In a previous PET study, Suzuki et al. observed significant hypermetabolism in the thalamus in these patients (77). Other studies have also reported hypermetabolism of glucose in the thalamus and striatum of patients with blepharospasm (78, 79). We hypothesized that thalamic activity is related to the etiology of blepharospasm and is worthy of in-depth exploration.

## 5 Neurotransmitter

Although the exact pathogenesis of blepharospasm has not been fully elucidated, some studies have shown that blepharospasm may be related to dysfunction in a variety of neurotransmitter systems, such as dopamine receptor hypersensitivity, cholinergic nervous system overactivity, γ-aminobutyric acid (GABA) dysfunction (4, 80–82) (Table 1).

**Table 1:** Related neurotransmitters and their functions of BSP.

**Neurotransmitter**	**Producing site**	**Excitatory/inhibitory neurotransmitters**	**Physiological function**
Dopamine	Substantia nigra cell	Excitability	Involved in neuropsychiatric activities
Serotonin	Raphe nucleus of brain stem	Inhibition	Involved in mood regulation, behavior management, sleep cycle maintenance
Acetylcholine (Ach)	• Brain stem: middle pontine tegmental region • Basal forebrain: Meinart's basal nucleus and medial septal nucleus	• Excitability (neuromuscular junction) • Inhibitory(cardiac vagal nerve terminal)	Participates in skeletal muscle contraction and maintain human consciousness
*GABA*	Brain and spinal cord	inhibition	Improves sleep, calms, lowers blood pressure

### 5.1 Dopamine

**Figure 1:**
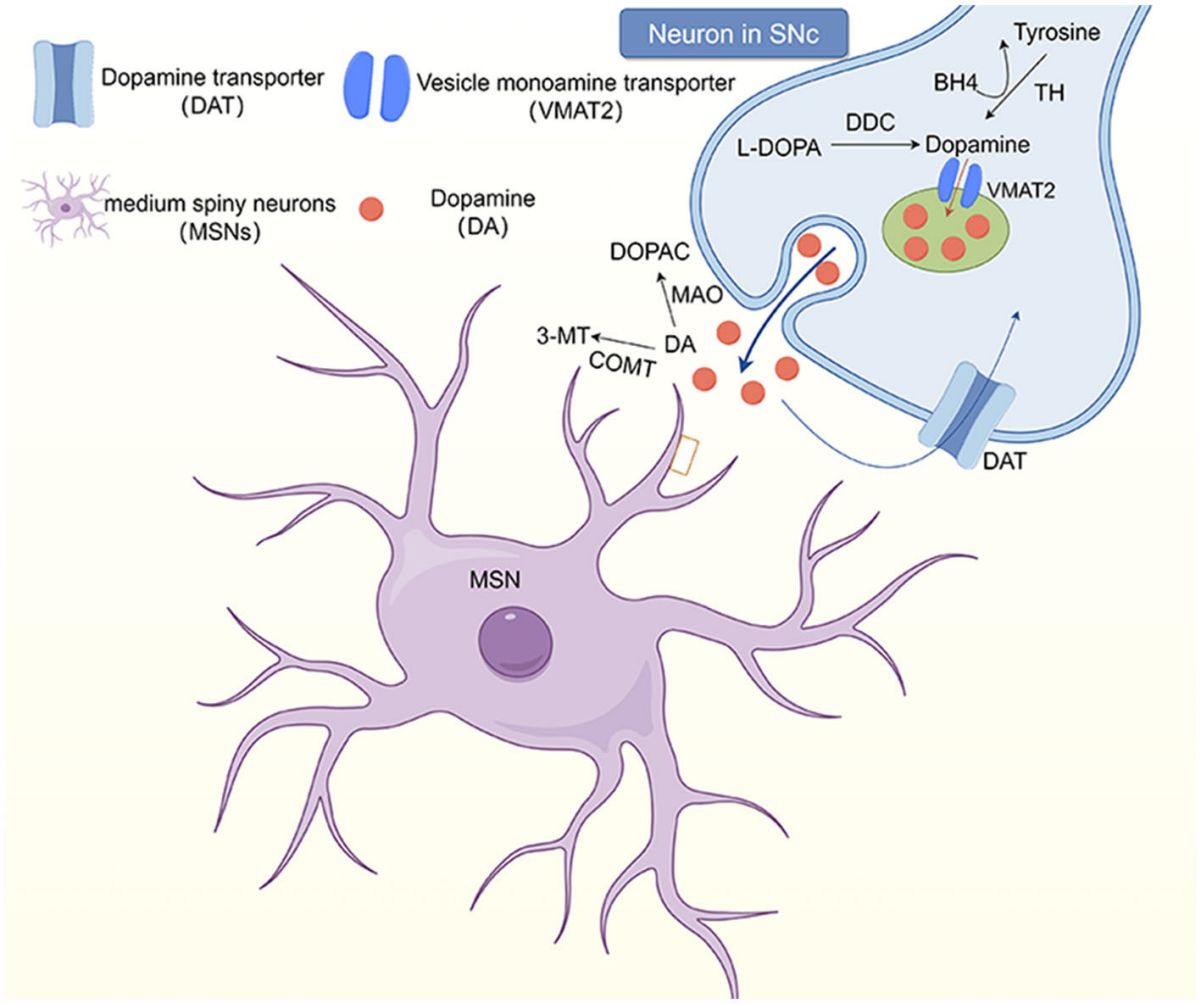
The dopaminergic system in the striatum. Neurons in the substantia nigra pars comoacta (SNc) receive dopaminergic input through the substantia striatum pathway. At the synaptic level, tyrosine hydroxylase (TH) uses tetrahydrobiopterin (BH4) as a cofactor to convert to tyrosine, which then synthesizes dopamine at the presynaptic terminal. Dopamine (DA) and other monoamines are introduced into vesicles at the presynaptic terminal by vesicular monoamine transporters (VMAT2). However, monoamines are released into the synaptic gap and bind to postsynaptic receptors, including dopamine receptors (D1-5). DA in the synaptic gap is degraded by monoamine oxidase and catechol-oxo-methyltransferase (COMT) to 3, 4-dihydroxyphenylacetic acid (DOPAC) and 3-methoxytyramine (3-MT), respectively, and transported back to the presynaptic terminal.

Dopamine (DA) is considered to be one of the main neurotransmitters involved in dystonia. It plays a very important role in maintaining muscle tone and regulating the voluntary movement of the human body. Subtle abnormalities in dopamine innervation are predisposing factors for blepharospasm, which may explain the greater occurrence in older adults with physiologically decreased dopamine (83). However, the exact mechanism through which the dopaminergic system is involved in the pathophysiology of dystonia remains unclear (84). It has been hypothesized that the cause of blepharospasm may be abnormalities in dopamine or other neurotransmitter metabolic pathways, caused by a reduction of dopamine receptors (Figure 1). For example, certain drugs, such as dopamine agonists (levodopa, bromocriptine, etc.), can induce blepharospasm (4, 82), because they cause hypofunction of the nigrostriatal GABA-ergic neurons, thereby causing dopaminergic receptor hypersensitivity, which increases central dopaminergic activity and thus causes cervicofacial dystonia.

Alterations in dopamine levels in the striatum may lead to imbalances in the direct and indirect striatal–pallidum pathways (85). The projection neuron (SPN) is the major cell type in the striatum and is the origin of the direct and indirect striatal–pallidum pathways. The SPN projects from the striatum to the external pallidum (GPe), internal (GPi) pallidum, and substantia nigra reticularis (SNr) regions. The projections from the SPN to the GPi and SNr are the “direct pathway,” while the projections to the GPe are the “indirect pathway.” Dopamine induces activation of the dopamine receptors D1R and D2R, which have opposing effects on the direct and indirect striatal–pallidum pathways. Hypermobility occurs when the direct striatal–pallidum pathway is adequately stimulated, whereas severe motor inability occurs when the indirect striatal–pallidum pathway is stimulated. Ribot et al. (84) proposed a putative model in which dopamine in the striatum explains different dystonia phenotypes by strictly controlling the balance between the direct and indirect striatal–pallidum pathways.

3-Methoxytyramine (3-MT), a metabolite of DA, is a newly recognized type of neuromodulator, which may be involved in motor control in some cases (86). The concentration of 3-MT in the brain is strictly controlled by the activities of catechol-O-methyltransferase (COMT) (87) and monoamine oxidase (MAO), mainly MAO-A (88, 89). An increase in COMT activity or a decrease in MAO activity leads to an increase in 3-MT concentration. The central COMT-mediated dopamine metabolite 3-MT affects exercise by interacting with the trace amine-related receptor 1 (89). We speculate that 3-MT elevation may be closely related to blepharospasm. The study conducted by Timmers et al. (85) observed higher 3-MT levels in patients with spasmodic torticollosis, dopa-responsive dystonia (DRD), and myoclonus-dystonia, compared to controls. The current study demonstrates alterations in the dopaminergic in patients with dystonia. Our study is also the first to demonstrate a 3-MT abnormality in patients with dystonia using plasma catecholamines, providing more comprehensive evidence for the pathogenesis of dystonia. Therefore, based on the available studies, we propose elevated 3-MT as a biomarker for any dystonia. In the future, patients with dystonia should be studied in greater depth.

### 5.2 Serotonin

In the central nervous system, serotonin is produced almost entirely by neurons in the raphe nucleus, located on the midline of the brain stem (90). Wallman et al. (91) found that the dorsal raphe nucleus (dRN) and its serotonergic neurons are connected to the basal ganglia and sensorimotor cortex. Serotonin, which is highly expressed in the limbic system, is a neurotransmitter of market interest, because it may be an emotion modulator that regulates neural activity and neuropsychological processes. Its role in psychiatric disorders has been extensively studied (92) and dysregulation of the serotonergic system is associated with the pathogenesis of many psychiatric and neurological disorders (90, 93, 94).

The serotonin system is related to dystonia in that levels of 5-hydroxyindoleacetic acid (the main metabolite of serotonin) are reduced in patients with dystonia (95). Timmers et al. found that patients with dystonia have significantly lower concentrations of tryptophan (a precursor of serotonin) than do healthy individuals (85). Currently, no effective treatment options for most forms of dystonia are available. Further research into selective serotonergic drugs may lead to the development of new treatments for dystonia.

### 5.3 Cholinergic nervous system

The interaction between dopamine and acetylcholine in the striatum is an important contributor to dystonia. It has been shown that acetylcholine has a powerful effect on striatal dopamine neurotransmission and plays a key role in motor learning (96). Acetylcholine agonists can induce blepharospasm, whereas anticholinergic drugs (e.g., benzedrine) can affect the release of dopamine in the striatum, thereby ameliorating blepharospasm. Much of the emerging literature emphasizes the centrality of cholinergic transmission in controlling voluntary movement and clinical manifestations of movement disorders (97).

### 5.4 GABA

Previous studies have suggested that dysfunction of GABA-A receptors in the striatum, primary and premotor cortex, somatosensory cortex, and cerebellum may be associated with the presentation of dystonia (98, 99). Clonazepam is a benzodiazepine receptor agonist that promotes the release and transmission of GABA, and long-term use of benzodiazepines can cause GABA receptors in the brain to be downregulated, sometimes resulting in blepharospasm (76). Suzuki et al. (100) suggested that abnormal GABA function may be associated with the occurrence of blepharospasm. Förstera et al. (101) found that ethanol enhanced GABAergic neurotransmission in the brain, which explains why alcohol consumption may reduce the risk of blepharospasm.

## 6 Conclusion

Blepharospasm is a condition that requires interdisciplinary understanding. Its etiology and pathogenesis are still not fully understood. Previous studies have suggested that blepharospasm is a functional disorder of the basal ganglia. However, recent studies have linked blepharospasm to motor network disorders. Further studies have also shown that it may be related to decreased GABA signaling in the nigra striatum, which results in inhibition of dopamine function, cholinergic hyperexcitability, and other neurotransmitter imbalances. Over the past few decades, the treatment of blepharospasm has greatly improved. The current first-line treatment for blepharospasm is Botox injection. Through this review, we boldly speculate that the increase in 3-MT in neurotransmitters is a biomarker of blepharospasm, which is worthy of further clinical research and exploration. This may provide a basis for the development of more effective therapeutic drugs and a guarantee for alleviating the clinical symptoms of blepharospasm and improving patients' quality of life.

## Author contributions

LZ: Conceptualization, Investigation, Software, Writing—original draft. HM: Investigation, Software, Supervision, Validation, Visualization, Writing—review & editing. WZ: Investigation, Supervision, Validation, Visualization, Writing—review & editing. WX: Investigation, Software, Supervision, Writing—review & editing. HS: Conceptualization, Software, Visualization, Writing—review & editing. SH: Conceptualization, Investigation, Supervision, Validation, Visualization, Writing—original draft.

## References

[1] Ma H Qu J Ye L Shu Y Qu Q. Blepharospasm, oromandibular dystonia, and meige syndrome: clinical and genetic update. Front Neurol. (2021) 12:630221. 10.3389/fneur.2021.630221 33854473 PMC8039296

[2] Hassell TJW Charles D. Treatment of blepharospasm and oromandibular dystonia with botulinum toxins. Toxins. (2020) 12. 10.3390/toxins12040269 32331272 PMC7232182

[3] Wakakura M Yamagami A Iwasa M. Blepharospasm in Japan: a clinical observational study from a large referral hospital in Tokyo. Neuroophthalmology. (2018) 42:275–83. 10.1080/01658107.2017.1409770 30258472 PMC6152494

[4] Pandey S Sharma S. Meige's syndrome: history, epidemiology, clinical features, pathogenesis and treatment. J Neurol Sci. (2017) 372:162–70. 10.1016/j.jns.2016.11.053 28017205

[5] Jahngir MU Ameer MA Patel BC. Meige syndrome. In: StatPearls. Treasure Island, FL: StatPearls Publishing, L. L. C. Copyright, © (2023). 30020730

[6] Peckham EL Lopez G Shamim EA Richardson SP Sanku S Malkani R. Clinical features of patients with blepharospasm: a report of 240 patients. Eur J Neurol. (2011) 18:382–6. 10.1111/j.1468-1331.2010.03161.x 20649903 PMC3934127

[7] Levey AS Eckardt KU Dorman NM Christiansen SL Hoorn EJ Ingelfinger JR. Nomenclature for kidney function and disease: report of a Kidney Disease: Improving Global Outcomes (KDIGO) Consensus Conference. Kidney Int. (2020) 97:1117–29. 10.1016/j.diabres.2020.108248 32409237

[8] Jochim A Meindl T Huber C Mantel T Zwirner S Castrop F. Treatment of blepharospasm and Meige's syndrome with abo- and onabotulinumtoxin A: long-term safety and efficacy in daily clinical practice. J Neurol. (2020) 267:267–75. 10.1007/s00415-019-09581-w 31630241

[9] Hallett M Evinger C Jankovic J Stacy M. Update on blepharospasm: report from the BEBRF International Workshop. Neurology. (2008) 71:1275–82. 10.1212/01.wnl.0000327601.46315.85 18852443 PMC2676990

[10] Sun Y Tsai PJ Chu CL Huang WC Bee YS. Epidemiology of benign essential blepharospasm: a nationwide population-based retrospective study in Taiwan. PLoS One. (2018) 13:e0209558. 10.1371/journal.pone.0209558 30586395 PMC6306223

[11] de Carvalho Aguiar PM Ozelius LJ. Classification and genetics of dystonia. Lancet Neurol. (2002) 1:316–25. 10.1016/S1474-4422(02)00137-0 12849429

[12] Ledoux MS Dauer WT Warner TT. Emerging common molecular pathways for primary dystonia. Mov Disord. (2013) 28:968–81. 10.1002/mds.25547 23893453 PMC3838975

[13] Evinger C. Benign essential blepharospasm is a disorder of neuroplasticity: lessons from animal models. J Neuroophthalmol. (2015) 35:374–9. 10.1097/WNO.0000000000000317 26576017

[14] Defazio G Hallett M Jinnah HA Conte A Berardelli A. Blepharospasm 40 years later. Mov Disord. (2017) 32:498–509. 10.1002/mds.26934 28186662 PMC5941939

[15] Lee JM Baek JS Choi HS Kim SJ Jang JW. Clinical features of benign essential blepharospasm in Korean patients. Korean J Ophthalmol. (2018) 32:339–43. 10.3341/kjo.2018.0038 30311455 PMC6182215

[16] Titi-Lartey OA Patel BC. Benign essential blepharospasm. In: StatPearls. Treasure Island, FL: StatPearls Publishing, L. L. C. Copyright © (2023). 32809668

[17] Dong H Luo Y Fan S Yin B Weng C Peng B. Screening gene mutations in Chinese patients with benign essential blepharospasm. Front Neurol. (2019) 10:1387. 10.3389/fneur.2019.01387 32038460 PMC6989602

[18] Defazio G Abbruzzese G Aniello MS Di Fede R Esposito M Fabbrini G. Eye symptoms in relatives of patients with primary adult-onset dystonia. Mov Disord. (2012) 27:305–7. 10.1002/mds.24026 22173654

[19] Lu R Huang R Li K Zhang X Yang H Quan Y. The influence of benign essential blepharospasm on dry eye disease and ocular inflammation. Am J Ophthalmol. (2014) 157:591–7.e591. 10.1016/j.ajo.2013.11.014 24269849

[20] Molloy A Williams L Kimmich O Butler JS Beiser I McGovern E. Sun exposure is an environmental factor for the development of blepharospasm. J Neurol Neurosurg Psychiatry. (2016) 87:420–4. 10.1136/jnnp-2014-310266 25904812

[21] Junker J Brandt V Berman BD Vidailhet M Roze E Weissbach A. Predictors of alcohol responsiveness in dystonia. Neurology. (2018) 91:e2020–6. 10.1212/WNL.0000000000006551 30341158 PMC6260199

[22] Imbriani P Sciamanna G El Atiallah I Cerri S Hess EJ Pisani A. Synaptic effects of ethanol on striatal circuitry: therapeutic implications for dystonia. FEBS J. (2022) 289:5834–49. 10.1111/febs.16106 34217152 PMC9786552

[23] Defazio G Martino D Abbruzzese G Girlanda P Tinazzi M Fabbrini G. Influence of coffee drinking and cigarette smoking on the risk of primary late onset blepharospasm: evidence from a multicentre case control study. J Neurol Neurosurg Psychiatry. (2007) 78:877–9. 10.1136/jnnp.2007.119891 17578856 PMC2117757

[24] Fisone G Borgkvist A Usiello A. Caffeine as a psychomotor stimulant: mechanism of action. Cell Mol Life Sci. (2004) 61:857–72. 10.1007/s00018-003-3269-3 15095008 PMC11138593

[25] Valls-Sole J Defazio G. Blepharospasm: update on epidemiology, clinical aspects, and pathophysiology. Front Neurol. (2016) 7:45. 10.3389/fneur.2016.00045 27064462 PMC4814756

[26] Berardelli A Rothwell JC Day BL Marsden CD. Pathophysiology of blepharospasm and oromandibular dystonia. Brain. (1985) 108 (Pt 3):593–608. 10.1093/brain/108.3.593 4041776

[27] Valls-Sole J. Assessment of excitability in brainstem circuits mediating the blink reflex and the startle reaction. Clin Neurophysiol. (2012) 123:13–20. 10.1016/j.clinph.2011.04.029 22030138

[28] Batla A Sánchez MC Erro R Ganos C Stamelou M Balint B. The role of cerebellum in patients with late onset cervical/segmental dystonia?–evidence from the clinic. Parkinson Relat Disord. (2015) 21:1317–22. 10.1016/j.parkreldis.2015.09.013 26385708

[29] Sharma N. Neuropathology of dystonia. Tremor Other Hyperkinet Mov. (2019) 9:569. 10.5334/tohm.514 PMC6420908 30886764

[30] Neychev VK Fan X Mitev VI Hess EJ Jinnah HA. The basal ganglia and cerebellum interact in the expression of dystonic movement. Brain. (2008) 131:2499–509. 10.1093/brain/awn168 18669484 PMC2724906

[31] Brüggemann N. Contemporary functional neuroanatomy and pathophysiology of dystonia. J Neural Trans. (2021) 128:499–508. 10.1007/s00702-021-02299-y 33486625 PMC8099808

[32] Khooshnoodi MA Factor SA Jinnah HA. Secondary blepharospasm associated with structural lesions of the brain. J Neurol Sci. (2013) 331:98–101. 10.1016/j.jns.2013.05.022 23747003 PMC3732185

[33] Kaji R Bhatia K Graybiel AM. Pathogenesis of dystonia: is it of cerebellar or basal ganglia origin? J Neurol Neurosurg Psychiatry. (2018) 89:488–92. 10.1136/jnnp-2017-316250 29089396 PMC5909758

[34] Girard B Davoudi O Tatry M Tassart M. Secondary blepharospasm, analysis and pathophysiology of blepharospasm. J Francais D'ophtalmol. (2021) 44:e1–12. 10.1016/j.jfo.2020.11.001 33349487

[35] Ross AH Elston JS Marion MH Malhotra R. Review and update of involuntary facial movement disorders presenting in the ophthalmological setting. Surv Ophthalmol. (2011) 56:54–67. 10.1016/j.survophthal.2010.03.008 21093885

[36] Hwang CJ Eftekhari K. Benign essential blepharospasm: what we know and what we don't. Int Ophthalmol Clin. (2018) 58:11–24. 10.1097/IIO.0000000000000207 29239874

[37] Quartarone A Sant'Angelo A Battaglia F Bagnato S Rizzo V Morgante F. Enhanced long-term potentiation-like plasticity of the trigeminal blink reflex circuit in blepharospasm. J Neurosci. (2006) 26:716–21. 10.1523/JNEUROSCI.3948-05.2006 16407569 PMC6674398

[38] Valls-Sole J. Spontaneous, voluntary, and reflex blinking in clinical practice. J Clin Neurophysiol. (2019) 36:415–21. 10.1097/WNP.0000000000000561 31688324

[39] Wilson BK Hess EJ. Animal models for dystonia. Mov Disord. (2013) 28:982–9. 10.1002/mds.25526 23893454 PMC3728703

[40] Zeuner KE Knutzen A Granert O Götz J Wolff S Jansen O. Increased volume and impaired function: the role of the basal ganglia in writer's cramp. Brain Behav. (2015) 5:e00301. 10.1002/brb3.301 25642386 PMC4309880

[41] Evinger C. Animal models for investigating benign essential blepharospasm. Curr Neuropharmacol. (2013) 11:53–8. 10.2174/157015913804999441 23814538 PMC3580792

[42] Ferrazzano G Conte A Fabbrini G Bologna M Macerollo A Defazio G. Botulinum toxin and blink rate in patients with blepharospasm and increased blinking. J Neurol Neurosurg Psychiatry. (2015) 86:336–40. 10.1136/jnnp-2014-307821 24963123

[43] Neychev VK Gross RE Lehéricy S Hess EJ Jinnah HA. The functional neuroanatomy of dystonia. Neurobiol Dis. (2011) 42:185–201. 10.1016/j.nbd.2011.01.026 21303695 PMC3478782

[44] Shakkottai VG. Physiologic changes associated with cerebellar dystonia. Cerebellum. (2014) 13:637–44. 10.1007/s12311-014-0572-5 24879387 PMC4156890

[45] Jinnah HA Neychev V Hess EJ. The anatomical basis for dystonia: the motor network model. Tremor Other Hyperkinet Mov. (2017) 7:506. 10.5334/tohm.383 29123945 PMC5673689

[46] Shakkottai VG Batla A Bhatia K Dauer WT Dresel C Niethammer M. Current opinions and areas of consensus on the role of the cerebellum in dystonia. Cerebellum. (2017) 16:577–94. 10.1007/s12311-016-0825-6 27734238 PMC5336511

[47] Tewari A Fremont R Khodakhah K. It's not just the basal ganglia: cerebellum as a target for dystonia therapeutics. Mov Disord. (2017) 32:1537–45. 10.1002/mds.27123 28843013 PMC5815386

[48] Mascia MM Dagostino S Defazio G. Does the network model fits neurophysiological abnormalities in blepharospasm? Neurol Sci. (2020) 41:2067–79. 10.1007/s10072-020-04347-z 32215851

[49] Poston KL Eidelberg D. Functional brain networks and abnormal connectivity in the movement disorders. Neuroimage. (2012) 62:2261–70. 10.1016/j.neuroimage.2011.12.021 22206967 PMC3489159

[50] Zhou B Wang J Huang Y Yang Y Gong Q Zhou D. A resting state functional magnetic resonance imaging study of patients with benign essential blepharospasm. J Neuroophthalmol. (2013) 33:235–40. 10.1097/WNO.0b013e31828f69e5 23636105

[51] Milardi D Quartarone A Bramanti A Anastasi G Bertino S Basile GA. The cortico-basal ganglia-cerebellar network: past, present and future perspectives. Front Syst Neurosci. (2019) 13:61. 10.3389/fnsys.2019.00061 31736719 PMC6831548

[52] Fagan M Scorr L Bernhardt D Hess EJ Perlmutter JS Pardo CA. Neuropathology of blepharospasm. Exp Neurol. (2021) 346:113855. 10.1016/j.expneurol.2021.113855 34464652 PMC8490317

[53] Prudente CN Pardo CA Xiao J Hanfelt J Hess EJ Ledoux MS. Neuropathology of cervical dystonia. Exp Neurol. (2013) 241:95–104. 10.1016/j.expneurol.2012.11.019 23195594 PMC3570661

[54] Hao X Huang X Yin X Wang HY Lu R Liang Z. Elucidation of the mechanism underlying impaired sensorimotor gating in patients with primary blepharospasm using prepulse inhibition. Front Neurol. (2023) 14:1105483. 10.3389/fneur.2023.1105483 36816573 PMC9929365

[55] Abbruzzese G Marchese R Buccolieri A Gasparetto B Trompetto C. Abnormalities of sensorimotor integration in focal dystonia: a transcranial magnetic stimulation study. Brain. (2001) 124:537–45. 10.1093/brain/124.3.537 11222454

[56] Hallett M. Neurophysiology of dystonia: the role of inhibition. Neurobiol Dis. (2011) 42:177–84. 10.1016/j.nbd.2010.08.025 20817092 PMC3016461

[57] Phukan J Albanese A Gasser T Warner T. Primary dystonia and dystonia-plus syndromes: clinical characteristics, diagnosis, and pathogenesis. Lancet Neurol. (2011) 10:1074–85. 10.1016/S1474-4422(11)70232-0 22030388

[58] Conte A Defazio G Hallett M Fabbrini G Berardelli A. The role of sensory information in the pathophysiology of focal dystonias. Nat Rev Neurol. (2019) 15:224–33. 10.1038/s41582-019-0137-9 30700825

[59] Desrochers P Brunfeldt A Sidiropoulos C Kagerer F. Sensorimotor control in dystonia. Brain Sci. (2019) 9:79. 10.3390/brainsci9040079 30979073 PMC6523253

[60] Baker RS Andersen AH Morecraft RJ Smith CD. A functional magnetic resonance imaging study in patients with benign essential blepharospasm. J Neuroophthalmol. (2003) 23:11–5. 10.1097/00041327-200303000-00003 12616082

[61] Martella G Tassone A Sciamanna G Platania P Cuomo D Viscomi MT. Impairment of bidirectional synaptic plasticity in the striatum of a mouse model of DYT1 dystonia: role of endogenous acetylcholine. Brain. (2009) 132:2336–49. 10.1093/brain/awp194 19641103 PMC2766181

[62] Peterson DA Sejnowski TJ Poizner H. Convergent evidence for abnormal striatal synaptic plasticity in dystonia. Neurobiol Dis. (2010) 37:558–73. 10.1016/j.nbd.2009.12.003 20005952 PMC2846420

[63] El Atiallah I Bonsi P Tassone A Martella G Biella G Castagno AN. Synaptic dysfunction in dystonia: update from experimental models. Curr Neuropharmacol. (2023) 21:2310–22. 10.2174/1570159X21666230718100156 37464831 PMC10556390

[64] Chiken S Nambu A. Disrupting neuronal transmission: mechanism of DBS? Front Syst Neurosci. (2014) 8:33. 10.3389/fnsys.2014.00033 24672437 PMC3954233

[65] Jiang W Lan Y Cen C Liu Y Feng C Lei Y. Abnormal spontaneous neural activity of brain regions in patients with primary blepharospasm at rest. J Neurol Sci. (2019) 403:44–9. 10.1016/j.jns.2019.06.002 31220741

[66] Xu J Luo Y Peng K Guo Y Zhong L Liu Y. Supplementary motor area driving changes of structural brain network in blepharospasm. Brain. (2023) 146:1542–53. 10.1093/brain/awac341 36130317

[67] Zhang M Huang X Li B Shang H Yang J. Gray matter structural and functional alterations in idiopathic blepharospasm: a multimodal meta-analysis of VBM and functional neuroimaging studies. Front Neurol. (2022) 13:889714. 10.3389/fneur.2022.889714 35734475 PMC9207395

[68] Horovitz SG Ford A Najee-Ullah MA Ostuni JL Hallett M. Anatomical correlates of blepharospasm. Transl Neurodegener. (2012) 1:12. 10.1186/2047-9158-1-12 23210426 PMC3514098

[69] Martino D Di Giorgio A D'Ambrosio E Popolizio T Macerollo A Livrea P. Cortical gray matter changes in primary blepharospasm: a voxel-based morphometry study. Mov Disord. (2011) 26:1907–12. 10.1002/mds.23724 21717508

[70] Fabbrini G Pantano P Totaro P Calistri V Colosimo C Carmellini M. Diffusion tensor imaging in patients with primary cervical dystonia and in patients with blepharospasm. Eur J Neurol. (2008) 15:185–9. 10.1111/j.1468-1331.2007.02034.x 18217887

[71] Berman BD Honce JM Shelton E Sillau SH Nagae LM. Isolated focal dystonia phenotypes are associated with distinct patterns of altered microstructure. NeuroImage Clin. (2018) 19:805–12. 10.1016/j.nicl.2018.06.004 30013924 PMC6024227

[72] Yang J Luo C Song W Guo X Zhao B Chen X. Diffusion tensor imaging in blepharospasm and blepharospasm-oromandibular dystonia. J Neurol. (2014) 261:1413–24. 10.1007/s00415-014-7359-y 24792726

[73] Yang J Luo C Song W Chen Q Chen K Chen X. Altered regional spontaneous neuronal activity in blepharospasm: a resting state fMRI study. J Neurol. (2013) 260:2754–60. 10.1007/s00415-013-7042-8 23900755

[74] Glickman A Nguyen P Shelton E Peterson DA Berman BD. Basal ganglia and cerebellar circuits have distinct roles in blepharospasm. Parkinson Relat Disord. (2020) 78:158–64. 10.1016/j.parkreldis.2020.06.034 32891945

[75] Guedj E Varrone A Boellaard R Albert NL Barthel H van Berckel B. EANM procedure guidelines for brain PET imaging using [(18)F]FDG, version 3. Eur J Nucl Med Mol Imaging. (2022) 49:632–51. 10.1007/s00259-021-05603-w 34882261 PMC8803744

[76] Suzuki Y Kiyosawa M Wakakura M Mochizuki M Ishiwata K Oda K. Glucose hypermetabolism in the thalamus of patients with drug-induced blepharospasm. Neuroscience. (2014) 263:240–9. 10.1016/j.neuroscience.2014.01.024 24462606

[77] Suzuki Y Mizoguchi S Kiyosawa M Mochizuki M Ishiwata K Wakakura M. Glucose hypermetabolism in the thalamus of patients with essential blepharospasm. J Neurol. (2007) 254:890–6. 10.1007/s00415-006-0468-5 17325818

[78] Galardi G Perani D Grassi F Bressi S Amadio S Antoni M. Basal ganglia and thalamo-cortical hypermetabolism in patients with spasmodic torticollis. Acta Neurol Scand. (1996) 94:172–6. 10.1111/j.1600-0404.1996.tb07049.x 8899050

[79] Esmaeli-Gutstein B Nahmias C Thompson M Kazdan M Harvey J. Positron emission tomography in patients with benign essential blepharospasm. Ophthal Plast Reconstr Surg. (1999) 15:23–7. 10.1097/00002341-199901000-00006 9949425

[80] Balint B Bhatia KP. Isolated and combined dystonia syndromes - an update on new genes and their phenotypes. Eur J Neurol. (2015) 22:610–7. 10.1111/ene.12650 25643588

[81] Zhao S Zhang Y Xu L Wei L Chen W. A case report of psychoactive drugs aggravating and alleviating Meige Syndrome. Shanghai Arch Psychiatry. (2016) 28:222–6. 10.11919/j.issn.1002-0829.216038 28638194 PMC5434272

[82] Kim JE Jung JW. Refractory dry eye disease associated with Meige's syndrome induced by long-term use of an atypical antipsychotic. BMC Ophthalmol. (2020) 20:474. 10.1186/s12886-020-01738-w 33267850 PMC7709279

[83] Quartarone A Ruge D. How many types of dystonia? Pathophysiological considerations. Front Neurol. (2018) 9:12. 10.3389/fneur.2018.00012 29527184 PMC5829064

[84] Ribot B Aupy J Vidailhet M Mazère J Pisani A Bezard E. Dystonia and dopamine: From phenomenology to pathophysiology. Prog Neurobiol. (2019) 182:101678. 10.1016/j.pneurobio.2019.101678 31404592

[85] Timmers ER van Faassen M Smit M Kuiper A Hof IH Kema IP. Dopaminergic and serotonergic alterations in plasma in three groups of dystonia patients. Parkinsonism Relat Disord. (2021) 91:48–54. 10.1016/j.parkreldis.2021.08.019 34482194

[86] Sotnikova TD Beaulieu JM Espinoza S Masri B Zhang X Salahpour A. The dopamine metabolite 3-methoxytyramine is a neuromodulator. PLoS ONE. (2010) 5:e13452. 10.1371/journal.pone.0013452 20976142 PMC2956650

[87] Solís O García-Montes JR Garcia-Sanz P Herranz AS Asensio MJ Kang G. Human COMT over-expression confers a heightened susceptibility to dyskinesia in mice. Neurobiol Dis. (2017) 102:133–9. 10.1016/j.nbd.2017.03.006 28315782 PMC5481205

[88] Myöhänen TT Männistö PT. Distribution and functions of catechol-O-methyltransferase proteins: do recent findings change the picture? Int Rev Neurobiol. (2010) 95:29–47. 10.1016/B978-0-12-381326-8.00003-X 21095458

[89] Espinoza S Manago F Leo D Sotnikova TD Gainetdinov RR. Role of catechol-O-methyltransferase (COMT)-dependent processes in Parkinson's disease and L-DOPA treatment. CNS Neurol Disord Drug Targets. (2012) 11:251–63. 10.2174/187152712800672436 22483291

[90] Berger M Gray JA Roth BL. The expanded biology of serotonin. Annu Rev Med. (2009) 60:355–66. 10.1146/annurev.med.60.042307.110802 19630576 PMC5864293

[91] Wallman MJ Gagnon D Parent M. Serotonin innervation of human basal ganglia. Eur J Neurosci. (2011) 33:1519–32. 10.1111/j.1460-9568.2011.07621.x 21375599

[92] Pauls DL Abramovitch A Rauch SL Geller DA. Obsessive-compulsive disorder: an integrative genetic and neurobiological perspective. Nat Rev Neurosci. (2014) 15:410–24. 10.1038/nrn3746 24840803

[93] Lin SH Lee LT Yang YK. Serotonin and mental disorders: a concise review on molecular neuroimaging evidence. Clin Psychopharmacol Neurosci. (2014) 12:196–202. 10.9758/cpn.2014.12.3.196 25598822 PMC4293164

[94] Timmers ER Plösch T Smit M Hof IH Verkaik-Schakel RN Tijssen MAJ. Methylation of the serotonin reuptake transporter gene and non-motor symptoms in dystonia patients. Clin Epigenet. (2022) 14:170. 10.1186/s13148-022-01384-7 36503539 PMC9743677

[95] Smit M Bartels AL van Faassen M Kuiper A Niezen-Koning KE Kema IP. Serotonergic perturbations in dystonia disorders-a systematic review. Neurosci Biobehav Rev. (2016) 65:264–75. 10.1016/j.neubiorev.2016.03.015 27073048

[96] Eskow Jaunarajs KL Bonsi P Chesselet MF Standaert DG Pisani A. Striatal cholinergic dysfunction as a unifying theme in the pathophysiology of dystonia. Prog Neurobiol. (2015) 127–8:91–107. 10.1016/j.pneurobio.2015.02.002 25697043 PMC4420693

[97] Bonsi P Cuomo D Martella G Madeo G Schirinzi T Puglisi F. Centrality of striatal cholinergic transmission in basal ganglia function. Front Neuroanat. (2011) 5:6. 10.3389/fnana.2011.00006 21344017 PMC3036975

[98] Garibotto V Romito LM Elia AE Soliveri P Panzacchi A Carpinelli A. *In vivo* evidence for GABA(A) receptor changes in the sensorimotor system in primary dystonia. Mov Disord. (2011) 26:852–7. 10.1002/mds.23553 21370265

[99] Marjańska M Lehéricy S Valabrègue R Popa T Worbe Y Russo M. Brain dynamic neurochemical changes in dystonic patients: a magnetic resonance spectroscopy study. Mov Disord. (2013) 28:201–9. 10.1002/mds.25279 23239076 PMC4410978

[100] Suzuki Y Ishii K Kiyosawa M. Role of GABAergic system in blepharospasm. J Neuroophthalmol. (2016) 36:349–50. 10.1097/WNO.0000000000000415 27533531

[101] Förstera B Castro PA Moraga-Cid G Aguayo LG. Potentiation of gamma aminobutyric acid receptors (GABAAR) by ethanol: how are inhibitory receptors affected? Front Cell Neurosci. (2016) 10:114. 10.3389/fncel.2016.00114 27199667 PMC4858537

